# Supervised Learning of Neural Networks for Active Queue Management in the Internet

**DOI:** 10.3390/s21154979

**Published:** 2021-07-22

**Authors:** Jakub Szyguła, Adam Domański, Joanna Domańska, Dariusz Marek, Katarzyna Filus, Szymon Mendla

**Affiliations:** 1 Faculty of Automatic Control, Electronics and Computer Science, Department of Distributed Systems and Informatic Devices, Silesian University of Technology, Akademicka 16, 44-100 Gliwice, Poland; adam.domanski@polsl.pl (A.D.); dariusz.marek@polsl.pl (D.M.); szymmen835@student.polsl.pl (S.M.); 2 Institute of Theoretical and Applied Informatics Polish Academy of Sciences, Bałtycka 5, 44-100 Gliwice, Poland; joanna@iitis.pl (J.D.); kfilus@iitis.pl (K.F.)

**Keywords:** neural networks, Hurst exponent, self-similarity, internet traffic, congestion control, dropping packets, active queue management, *PI^α^* controller

## Abstract

The paper examines the AQM mechanism based on neural networks. The active queue management allows packets to be dropped from the router’s queue before the buffer is full. The aim of the work is to use machine learning to create a model that copies the behavior of the AQM $PI^{\alpha }$ mechanism. We create training samples taking into account the self-similarity of network traffic. The model uses fractional Gaussian noise as a source. The quantitative analysis is based on simulation. During the tests, we analyzed the length of the queue, the number of rejected packets and waiting times in the queues. The proposed mechanism shows the usefulness of the Active Queue Management mechanism based on Neural Networks.

## 1. Introduction

Cisco predicts that by 2022, the Internet traffic will increase to 77 exabytes per month due to the rapid development of mobile technologies. The mobile data transfer will increase sevenfold compared to 2017, with an average annual growth of 46% [1]. The rapid increase in the number of Internet users as well as the transmission of multimedia content of increasing quality force the continuous development of data transmission mechanisms.

Wide area networks have their origins in the 1970s and were created for the American army. Thus, the most important aspect of the network based on a distributed architecture was to deliver reliable transmission of data and low connection costs. Unfortunately, the design assumptions proposed at the beginning turned out to be insufficient over the years.

Initially, IP routers handled packets according to the FIFO (First In First Out) rule (the first incoming packet in the queue is the first one to be served) [2]. For such scheduling, packets are dropped when the queue length exceeds the maximum length which results in the retransmission of a large number of packets in a short period of time. For such a network model, it is very difficult to control transmission throughput, delay and packet dropping [3].

To solve this problem, the Internet Engineering Task Force (IETF) proposed Active Queue Management (AQM) mechanisms [4]. These mechanisms preemptively drop packets before queue overflow occurs. In addition, the rejection of a packet should force the sender to reduce the transmission speed, which is provided by TCP congestion window mechanism [5]. The AQM algorithms used with TCP can enhance the efficiency of network transmission [4].

One of the first active queue management algorithm—Random Early Detection (RED) [6]—was proposed in 1993 by Sally Floyd and Van Jacobson. This mechanism estimates the packet dropping probability, which depends on the queue length. Despite the advantages of the RED algorithm, it also has some limitations. One of them is the problem of adjusting parameters to varying network traffic. Furthermore, the efficiency of the RED mechanism is closely related to the current network conditions [7]. There are many improvements and modifications of the classic RED algorithm [8,9,10,11,12,13] but none of them fully solves these problems. Performance of all RED family algorithms depends on coefficients of the dropping packet probability function. These coefficients should differ depending on the parameters of traffic such as intensity, burstiness or long-term dependence [14]. Article [15] presents the algorithm of finding the optimal parameters using the Hooke-Jeeves optimizing method. One of the newest solutions combines AQM mechanisms with a well-known method adopted from the theory of Automatic Control-PI controller. In this context, the information obtained from a classic PI controller is used as a packet dropping function [16,17,18]. The article [19] highlights the advantages of the PIE (Proportional Integral Enhanced Controller) algorithm. The authors state that mechanism easily adapts to varying transmission conditions and turned out to be a compromise between the degree of queue utilization and transmission delays.

The literature states that non-integer order controllers may have better performance than classic integer order ones. The first implementation of the fractional order PI controller used in queue management was presented in [20]. Our previous articles [21] investigate the performance of a fractional order PI controller ($PI^{\alpha }$) utilized as an Internet traffic controller.

Increase in popularity of machine learning methods may enable the creation of a more efficient AQM mechanism. Artificial Neural Networks (ANNs) are a powerful tool with high ability to recognize patterns, even in the case of incomplete and partially distorted training data [22]. One of their applications is time series processing and analysis, which is applied in many different fields. To process time-series data with Artificial Neural Networks, different types of network layers can be used, namely Recurrent layers (including Long-Short Term Memory (LSTM) layers and Gated Recurrent Unit (GRU) Layers) and 1D Convolutional Neural Networks (CNNs). Here, CNNs can be used as a fast alternative to recurrent layers [22]. Paper [23] proposes the CNN model for processing data from time series and forecasting prices in financial markets. In a different work, [24], CNNs were used to discover the network attacks, namely Distributed Denial of Service (DDoS) attacks. Additionally, our previous work [25] uses ANNs to examine the self-similar properties of the network traffic expressed by the Hurst parameter *H*. This approach also uses Convolutional Neural Networks. The promising results obtained in this work prompted us to create an efficient adaptive algorithm of Active Queue Management based on Convolutional Neural Networks.

Mechanisms that select AQM parameters based on the decisions of neural networks have been proposed in the literature [26,27]. Nevertheless, these methods are based on reinforcement learning. This paradigm relies on trial-and-error to make a specific decision in each iteration of the algorithm. The neural network receives feedback (i.e., queue length) after each step, which is then used to evaluate the previously made decision. Based on this feedback, the ANN changes its weights to optimize the accuracy of the decision-making process [28]. Thus, the configuration of the neural network varies depending on the current queue occupancy.

**Our contribution.** The aim of the work is to propose an algorithm for Active Queue Management based on supervised learning paradigm. We use a previously trained Convolutional Network to manage the queue. The ANN is trained based on the data obtained in simulations. We observe the impact on the behavior of the AQM mechanism based on the $PI^{\alpha }$ controller. In experiments we change the intensity and degree of self-similarity of network sources and observe behavior of the controller. The samples contain the sequence of incoming packets and the probability of packet dropping. The model trained this way is used as a new AQM mechanism. This paper presents its influence on the Internet transmission.

The remainder of the paper is organized as follows: Section 2 describes the current state of the art in this field. Section 3 presents the theoretical background. Section 4 is a description of the structure of the Artificial Neural Network, the data and the experimental methods used to obtain the results for this research. In Section 5 there is a description of the results of the conducted experiments. Section 6 concludes our research.

## 2. Related Works

There are many works regarding new AQM algorithms. These mechanisms are compared with existing solutions in terms of transmission parameters such as total number of dropped packets, average queue length, or transmission delays. In the article [7] passive and active queue management mechanisms were compared. Other works focus only on the comparison of the AQM mechanisms [6,29]. The topics of research in network and computer system performance evaluation also include works considering the impact of self-similarity of network traffic on transmission efficiency [10].

Additionally, the fractional order PI controller [30] is used for the Active Queue Management. This research is still under development, and its mechanisms have also been subjected to an analysis of the effect of the degree of self-similarity and long-term dependence of the traffic [31].

A separate group includes studies that have used neural networks to improve the queue management mechanism in TCP networks. The article [32] proposed the AQM mechanism based on reinforcement learning—Q-learning RED. The authors of [33] proposed an ANB-AQM mechanism, in which a back-propagation algorithm was used to train the neural networks to make decisions about accepting or rejecting packets. Article [34] proposes a neural network model, which modifies the REM algorithm, called the Fuzzy Neuron REM (FNREM) mechanism. This mechanism modifies the value of the proportional integral of the REM algorithm, by using the value of the proportional-integral derivative neuron as an indicator of overload.

ANNs were also used to create a new algorithm—Adaptive Neuron Proportional Integral Differential (ANPID) [35]. This mechanism used a single neuron to tune the PID controller coefficients. The authors of [36] presented the results based on the simulation and the real tests in the Linux Kernel, which resulted in the presentation of another adaptive modification of the PID controller using neural networks—the GRPID mechanism.

In article [37] authors presented an improved PID AQM/TCP system based on the network built using the Long Short-Term Memory (LSTM) layers (a specific type of a recurrent layer). It allows to predict queue length in the next step. They used Root Mean Square Error (RMSE) as a loss function. LSTM layers were also used to predict the occurrence of transmission overloads [38].

The research presented in XuIeee is an example of an attempt to use unsupervised learning to create a more efficient AQM mechanism. For that purpose, the Hebbian Learning rule is used and a new adaptive PHAQM algorithm is presented.

Bisoy and Pattnaik [39] used feed-forward neural network to create an AQM mechanism, namely FFNN-AQM. The network consisted of two input neurons, three neurons in a single hidden layer and the single output neuron.

Zhou et al. [40] also presented an adaptive AQM mechanism based on a single neuron whose weights were selected using reinforcement learning rules. The application of reinforcement learning was also used in [41] to build a mechanism to reduce transmission delays.

There are many works on the topic of AQMs based on neural networks. However, in these works, in contrast to our approach, the neural networks were mainly created using reinforcement learning. In addition, the research results did not consider the analysis of the effect of traffic self-similarity and long-term dependence on transmission efficiency.

## 3. Theoretical Background

Self-similarity is widely observed in nature, but the term itself was introduced by Mandelbrot in 1960s and it generally means that the portion of the whole object can be considered an image of the whole in a reduced scale. The object is self-similar, when it exhibits the same statistical properties independently of the scale. Mandelbrot described it on the example of the scaled coastlines, which also exhibited self-similarity. This property can also be used in the case of time-series analysis. The degree of self-similarity in this case determines whether Long-Range Dependence (LRD) and Short-Range Dependence (SRD) occur in data. These relationships were observed as early as the middle of the twentieth century, when Sir H. E. Hurst described the occurrence of long-range dependence based on the value of water level fluctuations in the Nile River. Although the terms of self-similarity and LRD are sometimes used interchangeably, they are not the same [42].

A continuous-time series Y(t) is exactly self-similar when the following condition is satisfied:

(1) $$Y\left(t\right)\overset{d}{=}a^{-H}Y\left(at\right),$$

for t≥0,a≥0 and 0<H<1. It results in the statistical invariability in different time scales. *H* is usually used to denote the Hurst exponent/parameter, which expresses the degree of self-similarity. The parameter can take values from range (0;1), and specific values represent:

- H∈(0;0.5): negative correlation—the LRD does not occur (the SRD occurs).
- H=0.5: no correlation.
- H∈(0.5;1): positive correlation—the LRD occurs.

It was first proven in [43] that actual network traffic exhibits self-similarity. This work provided the motivation for numerous studies that demonstrated the significant impact of self-similarity on TCP transmissions [44], or to confirm its occurrence in Wide Area Networks (WANs) [45]. Self-similarity results in performance degradations, such as mean queue length enlargement and the increase in packet loss probability [42]. The topic of self-similarity is still relevant in the literature and found its application in e.g., DoS attack detection (e.g., [46]). Our previous works were also related to this topic. They regarded determining the degree of traffic self-similarity expressed by the Hurst parameter and also using data obtained from the IITiS data traffic traces to examine self-similar properties [25]. Self-similarity significantly impacts queue occupancy and transmission performance [47]. For that reason, the samples generated for the purpose of this article are characterized by different degrees of self-similarity.

Artificial Neural Networks have found application in many different domains, e.g., image classification, natural language processing, signal processing etc. Additionally, Deep Learning approaches have become a solution to many problems due to their better ability to extract patterns than shallow learning [48]. The versatility of neural networks has resulted in them also being frequently used in the network traffic domain for tasks including attack detection [49,50], traffic generation [51] and classification of the traffic type [52].

Network traffic and its features are often represented as a time series. To process time-series data with Artificial Neural Networks, different types of networks (e.g., Autoencoders) and layers can be used, namely Recurrent layers (including Long-Short Term Memory (LSTM) layers and Gated Recurrent Unit (GRU) Layers) and 1D Convolutional Neural Networks.

Autoencoders can be built using different types of layers, e.g., Dense Layers or Convolutional layers. The goal of this type of network is to compress input data and then reconstruct it on output [53]. It can be used for the purpose of data denoising, but also anomaly detection. When the neural network is not able to reconstruct the input data well, it suggests that the sample can be anomalous [53].

LSTM layers are often used for the purpose of time-series data processing. Single LSTM units solve the gradient vanishing and exploding issues typical for simple Recurrent Layers and are able to propagate gradients over a long period of time [54]. The key characteristic of this type of layer is that they store the internal state, which enables them to ’remember’ the past information [52]. Due to that their internal ’memory’ is longer than in the traditional recurrent units.

The alternative for LSTM layers is a GRU layer. It is very similar to LSTM layer, also stores the Long-Time memory of the past information, which is vital for time-series processing. Nevertheless, it is simpler to implement and compute than the LSTM layer, thus more efficient [55].

Additionally, convolutional layers can be used to process time-series data. In this case, time has to be treated as a spatial dimension [22]. In fact, it is an efficient alternative to recurrent layers. In a Convolutional Neural Network, transformed time-series data are processed in turns using convolutional and pooling layers. As a result deep, more abstract representations are generated on the basis of raw data. Processing ends with a classifier part (Multi-Layer Perceptron), which consists of dense layers.

## 4. Data Preparation and Neural Networks Training Process

In this paper, we used artificial neural network models to develop an active queue management mechanism. The neural networks were trained to mimic the operation of the AQM based on the fractional order $PI^{\alpha }$ controller mechanism. The training data were generated based on simulation data, and a detailed description of the learning model is given in this section.

**Figure 1:**
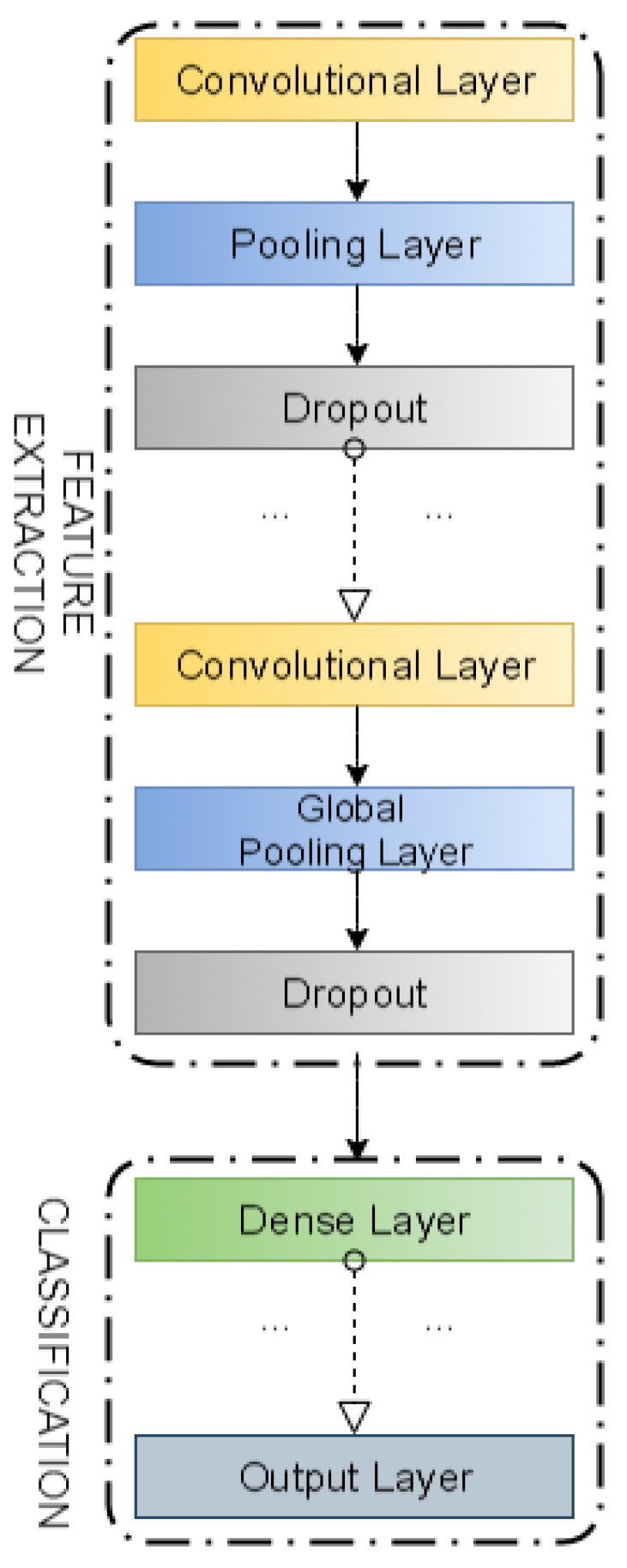
The conceptual structure of a Convolutional Neural Network based classifier used to model an Active Queue Management mechanism.

The neural network model was based on four convolutional layers and two dense layers. After each convolutional layer, the data were normalized and the results were averaged. Additionally, a dropout layer was placed to prevent over-fitting to the learning data. Python and Keras libraries were used to implement the model. The conceptual structure of the model used in this paper is presented in Figure 1. To design this model structure we relied on the experience of our earlier work [25], where the degree of self-similarity of network traffic was classified using Convolutional Neural Networks expressed by Hurst parameter.

In order to prepare the training set for the proposed neural network model, network simulations were performed, reflecting the queueing behavior of a fractional order controller $PI^{\alpha }$. The values of the fractional order $PI^{\alpha }$ controller parameters have been presented in the Table 1. These values were determined based on our previous work [26]. The results of these articles have shown that the choice of controller parameters significantly affects the queue length control properties. The process of choosing proper AQM/PI controller parameters is non-trivial. It has a significant impact on the packet dropping function (i.e., for an integral order α it can strengthen and accelerate the response of a controller). Properly selected AQM parameters should allow us to obtain adaptation to the changing transmission conditions and desired queue behavior. We discussed the influence of these parameters on queue behavior in papers [15]. The controller parameters were chosen in such a manner that controller $PI^{\alpha }1$ was the weakest controller, and controller $PI^{\alpha }3$ was the strongest one, which implies a large number of packet rejections and ease of maintaining the desired queue length.

To obtain training data for an AQM model based on Convolutional Networks, network simulations were performed using the AQM mechanism. For this purpose, the discrete event simulator SimPy (written in Python) was used. This software is available under the MIT License and has been used in our previous works regarding the evaluation of AQMs [21,26].

Our simulation model was a discrete model of a G/M/1/N queue. The simulation time was divided into discrete time intervals of length dt. Arrival of a packet was generated (or not) in a given time slot by a traffic source. The source of traffic was self-similar and based on Fractional Gaussian Noise (FGN) process. The advantages of such a source have been described previously in the articles [10,15,25].

All experiments considered different degrees of traffic self-similarity expressed using Hurst parameter. In experiments the Hurst parameter changed between H=0.5 (no correlation) and H=0.9 (high degree of LRD).

The input intensity coefficient was set to a constant value λ=0.5. Thus, the simulation packet source always had a constant intensity. Parameter μ represents the time of packet processing and dispatching (probability of taking a packet from the queue). Different values of this coefficient were used in the experiments. The parameter μ took values between μ=0.5 (moderately stressed system) to μ=0.15 (highly stressed system). This choice of simulation parameters allowed us to observe all properties of the AQM mechanism.

In our experiments, we considered different numbers of items from queue occupancy history taken into consideration in the samples used to train Convolutional Networks. For simplicity, we refer to this number of samples as ’CNN History’. This length corresponded to the number of time slots in the simulation model that were used as training data for the network. For example CNN=200 refers to 200*dt time intervals taken into consideration. Throughout this time, we observed the behavior of the AQM queue.

Thus, the training data consisted of:

1. Learning features:
  (a) The last *n* items from the queue’s occupancy history (CNN History).
  (b) History of packet rejections in *n* last queue states
    where n∈[20;100;200;300;400;500;1000].
2. Classes:
  (a) 11 labels that mapped the probability of packet rejection to the current transmission conditions, according to the principle shown in Table 2.

Therefore, we considered different lengths of queue occupancy history, because from the perspective of the router, which is a low resource device, minimizing the length of the history would be beneficial. In our study, we tried to determine the minimum acceptable length of *n* last items of the queue’s occupancy history.

**Figure 2:**
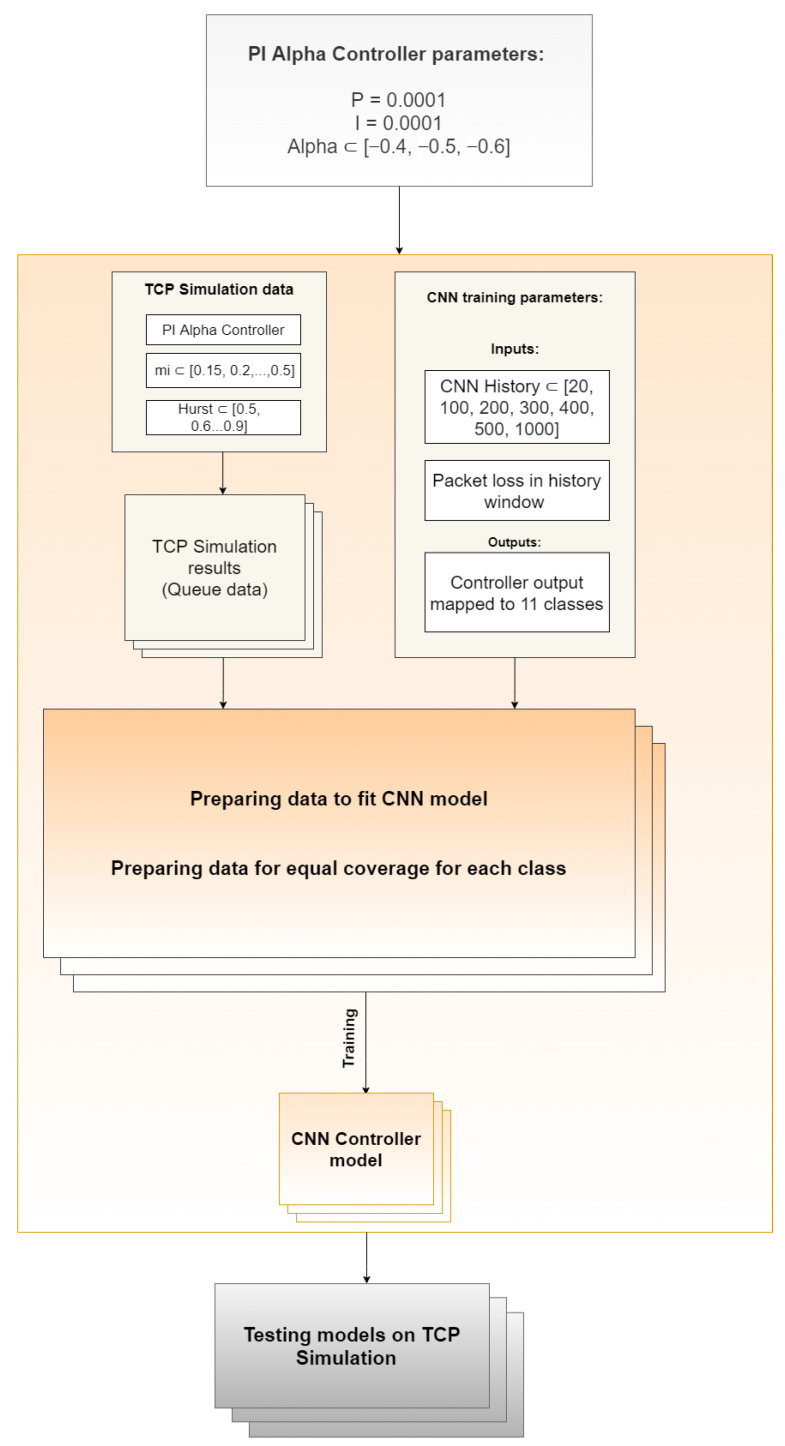
Training data preprocessing process.

For each probability interval, one million one-dimensional learning records were prepared. Therefore, the training set consisted of 11 million records. They contained transmission information such as the length of the queue in each consecutive time slot, the number of dropped packets, and the value of the $PI^{\alpha }$ controller’s packet rejection probability function. We present the process of data preparation in Figure 2. This amount of data seemed to be sufficient in comparison with the cardinality of data reported in the literature [56].

Input data prepared in such a manner were used in the process of supervised learning of the neural network models. In order to train the model and minimize the cost function, the optimizer Adaptive Moment Estimation (Adam) was used with the following parameters:

(2) $$\eta =10^{-3},\beta_{1}=0.9,\beta_{2}=0.999$$

where: η is the learning rate, $\beta_{1}$ is the exponential decay rate for the first moment estimates and $\beta_{2}$ is the exponential decay rate for the second moment estimates. The Adam optimizer is expressed by the equation [57]:

(3) $$\begin{array}{c} v_{t}=\beta_{1}v_{t-1}+\left(1-\beta_{1}\right)g_{t}\\ s_{t}=\beta_{2}s_{t-1}+\left(1-\beta_{2}\right)g_{t} \end{array}$$

where *v* is the first moment, which resembles momentum that records the past normalized gradient, *s* is the second moment and *g* denotes the gradient descent.

In both the four convolutional layers and the two dense layers, ReLU was used as the activation function and Sigmoid/Softmax functions were used to determine the activation of the output layer. Categorical cross-entropy was used as a cost function. Figure 1 shows the conceptual structure of a neural network model used for the purpose of active queue management mechanism.

We limited the training process to 10 epochs. This value was sufficient, since the values start to stabilize after only 5–6 epochs, as confirmed by the results in Table 3, Table 4 and Table 5. We also compare the accuracy of the model, when Softmax activation function (Table 3) and Sigmoid activation function (Table 4) were used in the output layer. Higher results were obtained for the Sigmoid function.

In the case of Softmax function (Table 3), the minimum accuracy was 32.3%, and the maximum 58.9%. For the models in which we applied the Sigmoid activation function for the last layer the minimum accuracy was 48.77% (for the network trained on the data from the $PI^{\alpha }3$ controller, where the CNN History =20), and the maximum 89.46% (for the network trained on the data from the $PI^{\alpha }3$ controller, where the CNN History =1000). Taking all the results into consideration, the best results were obtained for the CNN History ≥500, and the worst for the CNN History <100 (Table 4).

In the case of the model trained on data representing the behavior of three controllers simultaneously and the use of the Sigmoid activation function of the output layer, the maximum accuracy was 72.1% for the CNN History ≥500 (see Table 5).

## 5. Evaluation of the Neural Network-Based AQM

This section presents the behavior of the trained neural network (as assumed in Section 4 and evaluates its effectiveness as an AQM mechanism. This evaluation was performed using previously described simulation mechanisms. During the study, we evaluated the number of packets dropped from the queue and the average queue occupancy. We compared the effectiveness of the neural network-based AQM mechanism with the results of the $PI^{\alpha }$ controller-based AQM mechanism. We used the network traffic with different degrees of self-similarity during the experiments.

To increase the readability of the paper, we present only two extreme cases - the results obtained for a non-self-similar traffic (H=0.5) and for a traffic with high degree of LRD (H=0.9).

The intensity of the packet source in the simulation was assumed to be (lambda=0.5). On the other hand, the packet service time in a system was set to a constant value (μ=0.25) in order to obtain a heavily loaded system.

In our experiments, we evaluated four separate neural network models. The first three neural networks were trained with the data obtained from controllers $PI^{\alpha }1$, $PI^{\alpha }2$, and $PI^{\alpha }3$. The fourth model was trained with data regarding all of these controllers. In the first phase of the experiment, we considered two neural network models (see Figure 1): the first one with Softmax, and the second one with Sigmoid activation function of the last layer.

**Figure 3:**
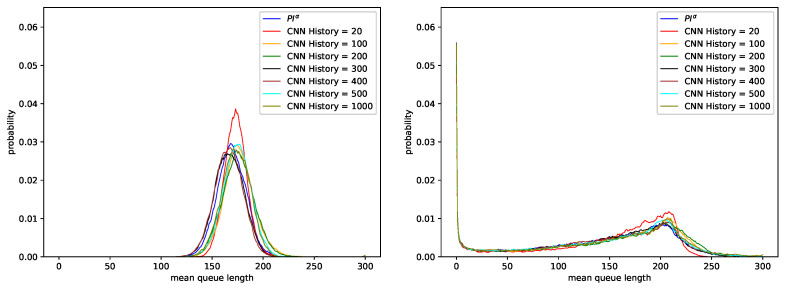
Distribution of queue length obtained for CNN model with the last layer activation function Sigmoid, trained using data regarding $PI^{\alpha }1$ controller and parameters: $K_{P}=0.0001$, $K_{I}=0.0004$, α=−0.4, H=0.5 (**left**), H=0.9 (**right**).

**Figure 4:**
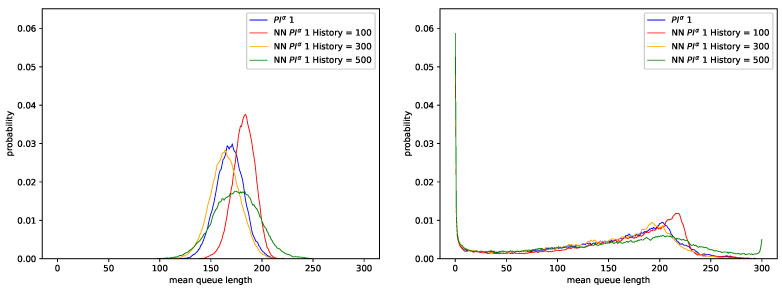
Distribution of queue length obtained for CNN model with the last layer activation function Softmax, trained using data regarding $PI^{\alpha }1$ controller and parameters: $K_{P}=0.0001$, $K_{I}=0.0004$, α=−0.4, H=0.5 (**left**), H=0.9 (**right**).

A comparison of Table 3 and Table 4 shows that although Softmax function is more commonly used in the literature as an activation function of the output layer of the neural network for multiclass classification, Sigmoid function performs better in our case. In the worst case, in which the network obtained accuracy of 32.31%, changing the activation function to Sigmoid resulted in significant accuracy increase (65.65%). Additionally, in the best obtained case accuracy changed from 58.90% to 86.65%. Figure 3 and Figure 4 show average queue lengths for AQM mechanism based on neural network. Detailed results are compared on Table 6 and Table 7 for Sigmoid function and on Table 8 and Table 9 for Softmax function. Both presented networks imitate the behavior of the first controller—$PI^{\alpha }$ (see Table 1). Comparing the number of discarded packets and the average queue sizes, we find that they are similar regardless of the chosen network activation function in the last layer. As Hurst increases, the number of dropped packets decreases slightly in the case of Sigmoid function (<1%).

Taking into consideration higher accuracy obtained using Sigmoid function, we chose this function to be used in further experiments.

Figure 3 compares the behavior of two AQM mechanisms: $PI^{\alpha }1$ controller and the CNN-based AQM trained on the data reflecting the behavior of this controller.

For the CNN model, different lengths of the last *n* elements of the queue occupancy history (input to the neural network) were considered. Regardless of the value of *n*, the resulting queue length distributions are similar to the queue length distribution of the $PI^{\alpha }1$ controller. For Poisson traffic (non-self-similar traffic, H=0.5), the average queue length oscillates between 166 and 176 packets (see Table 6). For highly self-similar traffic (parameter H=0.9), the average queue length was between 139 and 147 (see Table 7). In this case, all the Convolutional Neural Network models (with different numbers of CNN History) obtained larger values of the average queue length, with fewer packets dropped, than the $PI^{\alpha }1$ mechanism.

**Figure 5:**
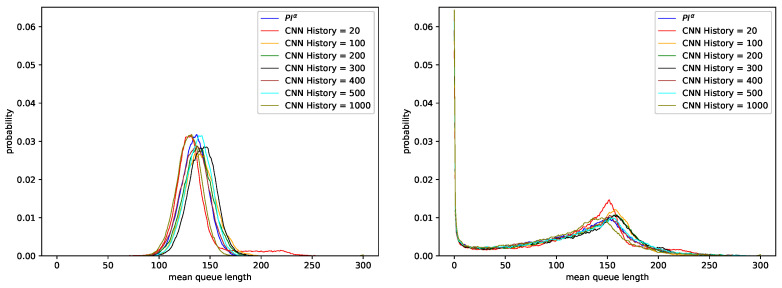
Distribution of queue length obtained for CNN model with the last layer activation function Sigmoid, trained using data regarding $PI^{\alpha }2$ controller and parameters: $K_{P}=0.0001$, $K_{I}=0.0004$, α=−0.5, H=0.5 (**left**), H=0.9 (**right**).

Figure 5 presents the results for stronger AQM mechanism $PI^{\alpha }2$. The detailed results of dropped packets numbers and queue lengths are presented in Table 10 and Table 11. Because of the fact that the $PI^{\alpha }2$ controller was stronger than the one presented above, the obtained average queue lengths were smaller.

**Figure 6:**
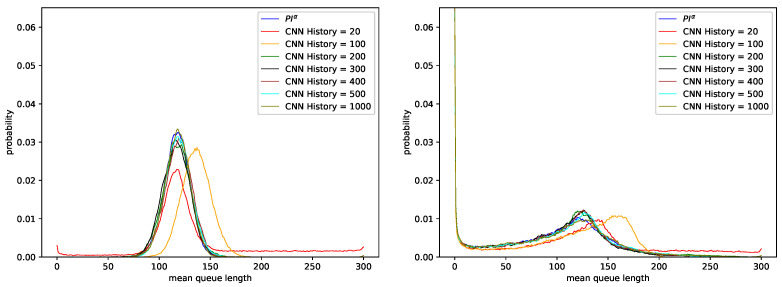
Distribution of queue length obtained for CNN model with the last layer activation function Sigmoid, trained using data regarding $PI^{\alpha }3$ controller and parameters: $K_{P}=0.0001$, $K_{I}=0.0004$, α=−0.6, H=0.5 (**left**), H=0.9 (**right**).

Figure 6 compares the last pair of controllers: controller $PI^{\alpha }3$, with the corresponding models based on Convolutional Neural Networks. The results prove that this controller is the strongest one. The AQM mechanism increased considerably the number of dropped packets and decreased the obtained queue lengths. In the case of traffic without LRD (see Table 12, for parameter H=0.5) the average queue occupancy oscillateds between 116 and 139 packets, and in the case of traffic characterized by a high degree of LRD (see Table 13, for parameter H=0.9) between 94 and 121 packets.

It should be noted that for all three CNN-based AQM models, a more efficient AQM model was obtained compared to the controllers that were used to create the test data. Even for the model that obtained the smallest accuracy during the learning process (48.77%, see Table 4), based on non-integer controller data of order $PI^{\alpha }3$, for CNN History <100, the obtained average queue length was larger than for the base mechanism $PI^{\alpha }3$. This situation occurred both for traffic without LRD (see Table 12) and for traffic characterized by a high degree of LRD (see Table 13).

**Figure 7:**
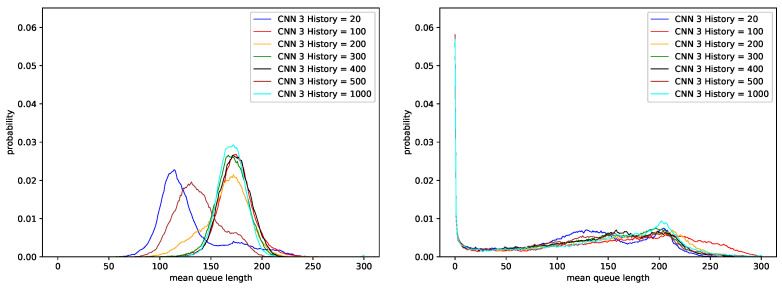
Distribution of queue length obtained for CNN controller trained using data regarding three $PI^{\alpha }$ controllers, H=0.5 (**left**), H=0.9 (**right**).

**Figure 8:**
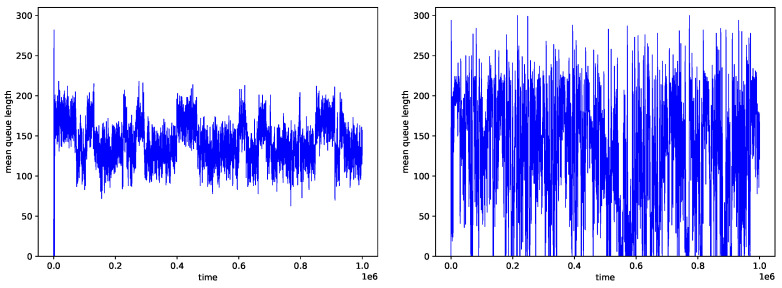
Queue occupancy obtained for CNN controller trained using data regarding three $PI^{\alpha }$ controllers, with CNN History =500, H=0.5 (**left**), H=0.9 (**right**).

In the next simulation step, we evaluated the AQM-CNN mechanism whose learning data were generated from the behavior of all three $PI^{\alpha }$ controllers. Figure 7 shows the queue distribution, and Figure 8 shows the changes in queue occupancy over time. Details of the number of packets dropped and the resulting average queue occupancy are presented in Table 14 for the traffic without LRD and in Table 15, for traffic with a high degree of LRD.

The results show that when the number of last *n* elements of queue occupancy history taken as a CNN input is too small (CNN History <100), then, independent of the degree of self-similarity of the traffic, the number of dropped packets, and the average queue length, approximates the results obtained using the sets of controllers $PI^{\alpha }2$ and $PI^{\alpha }3$ (see Table 10, Table 11, Table 12 and Table 13).

On the other hand, when the considered number of last *n* queue occupancy history elements is larger (CNN History ≥100), the obtained average queue length increases by 46 packets for traffic without LRD (Table 14, for H=0.5), or by 32 packets, for traffic characterized by a high degree of LRD (see Table 15, for H=0.9). This means that the resulting queue distribution matches the one of the original and the most efficient controller $PI^{\alpha }1$ (Figure 3).

This feature indicates that for the AQM model based on Convolutional Networks, as the number of story elements used increases, the ability of the mechanism to adapt to current Internet transmission conditions also improves.

## 6. Conclusions

The paper presents a new Active Queue Management mechanism based on Convolutional Neural Networks and supervised learning.

To train the Convolutional Networks used in the experiments, data obtained through simulation have been used. The training data of the CNN model reflect the behavior of the AQM mechanism, based on a fractional order controller $PI^{\alpha }$.

In our experiments, we took into account the effect of the degree of traffic self-similarity and long-term dependence on the performance of the proposed mechanism.

We also considered the effect of the number of last *n* elements of the queue occupancy history, used as input of the neural network, on the efficiency of the proposed mechanism. The best results were obtained for CNN History = 500. The minimum length of CNN History for which results are still acceptable is 100.

In the experiments, neural networks with different number of convolutional layers and different optimizers and cost functions were considered to build the AQM model. After comparing the results obtained with different activation functions, the results have shown that the most efficient model used Sigmoid activation function in the output layer, therefore we chose this function for further experiments. The decisions made in this work were also influenced by our previous work regarding traffic classification in terms of the degree of self-similarity [25].

The most efficient AQM obtained in our study was based on the Convolutional Neural Network model, trained using the data reflecting the behavior of all three $PI^{\alpha }$ controllers jointly.

The results confirmed that the model based on Convolutional Neural Networks can effectively reproduce the results of the classical AQM algorithm and effectively manage the data transmission. Such a model maintains the assumed average number of packets in the queue and reduces the total number of dropped packets, independent of the degree of traffic self-similarity.

It seems that the proposed mechanism exhibits some advantages over previously proposed mechanisms encountered in the literature. Our previous study [26] demonstrated that the reinforcement learning methods are well suited for maintaining the assumed queue size. However, in computer networks, the process of controlling packet traffic is more complex. The objective is to maximize the transmission efficiency. This efficiency is characterized by: throughput, delay, and possible retransmissions. Efficiency of AQM mechanisms is influenced by self-similarity of network traffic. The higher the Hurst parameter value is, the greater problems with correct packet management occur. The proposed solution addresses this problem much more effectively. The biggest disadvantage of this solution is greater computational and memory complexity of solutions based on Convolutional Neural Networks. This complexity may affect the difficulty of implementing this solution in real routers.

In our previous study [58], we used a Linux-based computer as a router. In that study, we used a special router implementation based on a special forwarding mechanism (based on the iptables mechanism), which delivered all packets to the user program implementing AQM. This solution greatly simplifies the research model. Unfortunately, the tests have shown that forwarding packets from kernel space to userspace requires a significant amount of time and is not optimal. In the target solutions the whole implementation should be realized in the kernel of the system. The implementation may be a great challenge on routers with low hardware resources. In such solutions instead of multiplication operations bit shifting is used, which causes calculation errors. For CNN calculations these errors may be too high. However, it seems that the computational power of routers will increase in the future. We want to devote a separate article to the problems of implementing AQMs in real routers.

## Figures and Tables

**Table 1:** The $PI^{\alpha }$ controller parameters.

	$K_{P}$	$K_{I}$	α
$PI^{\alpha }1$	0.0001	0.0004	−0.4
$PI^{\alpha }2$	0.0001	0.0004	−0.5
$PI^{\alpha }3$	0.0001	0.0004	−0.6

**Table 2:** Decision class labels representing ranges of probabilities of packet being dropped.

Decision Class	Probability Interval [%]
1	[0;5)
2	[5;15)
3	[15;25)
4	[25;35)
5	[35;45)
6	[45;55)
7	[55;65)
8	[65;75)
9	[75;85)
10	[85;95)
11	[95;100]

**Table 3:** The accuracy measurements for testing the CNN model trained on data regarding three $PI^{\alpha }1$, $PI^{\alpha }2$ and $PI^{\alpha }3$ controllers, *n* last items in queue occupancy history taken into consideration (we used Softmax function as an activation function of the last layer).

		Softmax						
		*n* History Length						
		20	100	200	300	400	500	1000
**CNN by behavior ${\mathrm{PI}}^{\alpha }1$**	5 epochs	52.27	54.48	54.50	56.67	58.40	58.81	51.59
	6 epochs	52.36	54.88	54.68	56.70	58.42	58.84	51.72
	10 epochs	52.40	55.50	54.83	56.74	58.47	58.90	51.82
**CNN by behavior ${\mathrm{PI}}^{\alpha }2$**	5 epochs	48.37	47.23	40.29	41.99	43.68	44.06	43.94
	6 epochs	48.45	47.61	40.32	42.06	43.74	44.06	44.00
	10 epochs	48.54	48.42	40.31	42.30	43.78	44.24	44.04
**CNN by behavior ${\mathrm{PI}}^{\alpha }3$**	5 epochs	47.07	41.83	33.80	32.30	32.92	33.79	38.45
	6 epochs	47.18	42.09	33.81	32.31	32.93	33.82	38.44
	10 epochs	47.41	42.62	34.18	32.32	32.92	33.83	38.50

**Table 4:** The accuracy measurements for test data for a neural network model trained with data representing the behavior of $PI^{\alpha }1$, $PI^{\alpha }2$ and $PI^{\alpha }3$ controllers, *n* last items in queue occupancy history taken into consideration (we used Sigmoid function as an activation function of the last layer).

		Sigmoid						
		*n* Last Items in Queue Occupancy History						
		20	100	200	300	400	500	1000
**CNN by behavior ${\mathrm{PI}}^{\alpha }1$**	5 epochs	55.98	73.95	80.76	83.67	85.22	86.15	88.46
	6 epochs	56.04	74.06	80.88	83.80	85.39	86.31	88.74
	10 epochs	56.16	74.38	81.19	84.13	85.69	86.64	89.46
**CNN by behavior ${\mathrm{PI}}^{\alpha }2$**	5 epochs	50.34	70.70	75.94	76.92	77.03	77.20	83.71
	6 epochs	50.38	70.83	76.08	77.06	77.18	77.32	84.04
	10 epochs	50.53	71.13	76.40	77.36	77.57	77.79	84.81
**CNN by behavior ${\mathrm{PI}}^{\alpha }3$**	5 epochs	48.77	67.16	67.54	65.65	64.39	64.04	77.46
	6 epochs	48.82	67.29	67.70	65.83	64.57	64.24	77.76
	10 epochs	49.06	67.60	68.03	66.23	64.99	64.68	78.68

**Table 5:** The accuracy measurements for test data for a neural network model trained with data representing the behavior of three controllers simultaneously, given *n* recent queue occupancy history elements.

		Sigmoid						
		*n* Last Items in Queue Occupancy History						
		20	100	200	300	400	500	1000
**CNN by behavior 3 ${\mathrm{PI}}^{\alpha }$**	5 epochs	49.52	67.03	68.55	68.48	68.40	68.42	70.98
	6 epochs	49.58	67.12	67.70	68.65	68.60	68.61	71.30
	10 epochs	49.74	67.34	68.99	69.03	69.15	69.21	72.10

**Table 6:** Detailed results of queue occupancy obtained for CNN model with the last layer activation function Sigmoid, trained using data regarding $PI^{\alpha }1$ controller and parameters: $K_{P}=0.0001$, $K_{I}=0.0004$, α=−0.4 and H=0.5.

AQM	Packet Dropped	Average Queue Length
$PI^{\alpha }1$	249,878	168.98
CNN History = 20	251,198	172.97
CNN History = 100	248,936	176.45
CNN History = 200	250,063	175.69
CNN History = 300	249,510	166.87
CNN History = 400	250,104	166.23
CNN History = 500	250,800	174.31
CNN History = 1000	249,561	173.33

**Table 7:** Detailed results of queue occupancy obtained for CNN model with the last layer activation function Sigmoid, trained using data regarding $PI^{\alpha }1$ controller and parameters: $K_{P}=0.0001$, $K_{I}=0.0004$, α=−0.4 and H=0.9.

AQM	Packet Dropped	Average Queue Length
$PI^{\alpha }1$	263,387	139.16
CNN History = 20	261,678	145.66
CNN History = 100	262,304	145.81
CNN History = 200	262,518	147.22
CNN History = 300	262,935	139.28
CNN History = 400	263,872	140.89
CNN History = 500	263,440	142.22
CNN History = 1000	263,654	143.14

**Table 8:** Detailed results of queue occupancy obtained for CNN model with the last layer activation function Softmax, trained using data regarding $PI^{\alpha }1$ controller and parameters: $K_{P}=0.0001$, $K_{I}=0.0004$, α=−0.4 and H=0.5.

AQM	Packet Dropped	Average Queue Length
$PI^{\alpha }1$	249,878	168.98
CNN History = 100	250,038	182.21
CNN History = 300	250,455	164.06
CNN History = 500	250,017	174.73

**Table 9:** Detailed results of queue occupancy obtained for CNN model with the last layer activation function Softmax, trained using data regarding $PI^{\alpha }1$ controller and parameters: $K_{P}=0.0001$, $K_{I}=0.0004$, α=−0.4 and H=0.9.

AQM	Packet Dropped	Average Queue Length
$PI^{\alpha }1$	263,387	139.16
CNN History = 100	261,271	148.89
CNN History = 300	263,609	134.64
CNN History = 500	264,569	145.64

**Table 10:** Detailed results of queue occupancy obtained for CNN model with the last layer activation function Sigmoid, trained using data regarding $PI^{\alpha }2$ controller parameters: $K_{P}=0.0001$, $K_{I}=0.0004$, α=−0.5 and H=0.5.

AQM	Packet Dropped	Average Queue Length
$PI^{\alpha }2$	250,314	134.72
CNN History = 20	249,610	135.27
CNN History = 100	250,657	140.37
CNN History = 200	249,633	137.95
CNN History = 300	249,752	142.29
CNN History = 400	248,852	134.86
CNN History = 500	249,960	138.85
CNN History = 1000	249,744	129.60

**Table 11:** Detailed results of queue occupancy obtained for CNN model with the last layer activation function Sigmoid, trained using data regarding $PI^{\alpha }2$ controller parameters: $K_{P}=0.0001$, $K_{I}=0.0004$, α=−0.5 and H=0.9.

AQM	Packet Dropped	Average Queue Length
$PI^{\alpha }2$	264,819	109.37
CNN History = 20	263,014	117.57
CNN History = 100	264,135	112.98
CNN History = 200	264,217	115.69
CNN History = 300	264,668	116.24
CNN History = 400	265,538	110.05
CNN History = 500	264,533	112.47
CNN History = 1000	265,839	105.25

**Table 12:** Detailed results of queue occupancy obtained for CNN model with the last layer activation function Sigmoid, trained using data regarding $PI^{\alpha }3$ controller and parameters: $K_{P}=0.0001$, $K_{I}=0.0004$, α=−0.6 and H=0.5.

AQM	Packet Dropped	Average Queue Length
$PI^{\alpha }3$	250,840	117.53
CNN History = 20	251,892	139.26
CNN History = 100	250,362	136.35
CNN History = 200	248,878	117.67
CNN History = 300	250,533	116.40
CNN History = 400	250,011	118.85
CNN History = 500	250,166	118.67
CNN History = 1000	249,801	118.20

**Table 13:** Detailed results of queue occupancy obtained for CNN model with the last layer activation function Sigmoid, trained using data regarding $PI^{\alpha }3$ controller and parameters: $K_{P}=0.0001$, $K_{I}=0.0004$, α=−0.6 and H=0.9.

AQM	Packet Dropped	Average Queue Length
$PI^{\alpha }3$	265,707	95.13
CNN History = 20	265,737	121.19
CNN History = 100	263,952	110.79
CNN History = 200	265,383	97.28
CNN History = 300	266,295	94.09
CNN History = 400	266,366	95.79
CNN History = 500	265,592	97.31
CNN History = 1000	266,184	96.90

**Table 14:** Detailed results of queue occupancy results obtained for CNN model trained using data regarding three $PI^{\alpha }$ controllers and H=0.5.

AQM	Packet Dropped	Average Queue Length
CNN 3 History = 20	249,727	128.75
CNN 3 History = 100	249,988	174.64
CNN 3 History = 200	250,583	164.87
CNN 3 History = 300	249,907	169.98
CNN 3 History = 400	249,593	173.41
CNN 3 History = 500	250,157	138.08
CNN 3 History = 1000	249,334	170.46

**Table 15:** Detailed results of queue occupancy results obtained for CNN model trained using data regarding three $PI^{\alpha }$ controllers and H=0.9.

AQM	Packet Dropped	Average Queue Length
CNN 3 History = 20	262,841	120.94
CNN 3 History = 100	263,859	152.15
CNN 3 History = 200	262,298	137.21
CNN 3 History = 300	263,205	131.49
CNN 3 History = 400	263,818	129.81
CNN 3 History = 500	263,872	127.37
CNN 3 History = 1000	263,554	138.32

## Data Availability

Not applicable.
